# Optimizing robotic approach to ventral hernia repair: an updated systematic review and meta-analysis between preperitoneal versus retromuscular repair

**DOI:** 10.1007/s11701-025-03076-9

**Published:** 2025-12-22

**Authors:** Francesco Brucchi, Sara Lauricella, Sofia Esposito, Gianpaolo Formisano, Roberto Cirocchi, Richard Sassun, Gianlorenzo Dionigi

**Affiliations:** 1 University of Milan, Via Festa del Perdono, 7, Milan, 20122 Italy; 2 Colorectal Surgery Unit, Fondazione IRCCS Istituto Nazionale Dei Tumori, Milan, 20133 Italy; 3 Department of General, Emergency Surgery and New Technologies, Baggiovara General Hospital, AOU Modena, Modena, Italy; 4 Department of Surgery, Dipartimento di Scienze della Salute, Asst Santi Paolo e Carlo, University of Milan, Milan, Italy; 5 Department of Digestive and Emergency Surgery, “S. Maria” Hospital, Terni, 05100 Italy; 6 Department of Medicine and Surgery, University of Perugia, Perugia, 06129 Italy; 7 Division of Surgery, Istituto Auxologico Italiano, Istituto di Ricovero e Cura a Carattere Scientifico (IRCCS), Milan, Italy; 8 Department of Pathophysiology and Transplantation, University of Milan, Milan, Italy

**Keywords:** Robotic ventral hernia repair, Abdominal wall surgery, Extraperitoneal repair, Preperitoneal repair, Minimally invasive surgery, Retromuscular repair, Systematic review, Meta-analysis

## Abstract

Background: The Rives–Stoppa retromuscular repair remains the reference standard for open ventral hernia repair. Advances in robotic surgery have expanded the minimally invasive options for extraperitoneal mesh placement, enabling both retromuscular and preperitoneal approaches. The robotic ventral transabdominal preperitoneal repair (Rv-TAPP), has seen increasing adoption, but comparative evidence with the robotic retromuscular techniques remains limited. Methods: A systematic review and meta-analysis were reported in accordance with PRISMA and AMSTAR II guidelines (PROSPERO 2025: CRD420251173188). Searches of PubMed, Embase, Scopus, and Cochrane Library were searched to identify comparative studies evaluating robotic preperitoneal and retromuscular ventral hernia repair. Random-effects models (DerSimonian–Laird) were used to pool weighted mean differences (WMD) and risk ratios (RR) with 95% confidence intervals (CI). Results: Six retrospective studies including 3704 patients (2326 preperitoneal, 1378 retromuscular) were included. Preperitoneal cases involved smaller, predominantly primary defects (mean 3.0 cm vs 6.3 cm). Operative time was significantly shorter for Rv-TAPP (WMD = −39.4 min, 95% CI −58.5 to −20.2; p< 0.001). Overall postoperative complications occurred in 7.2% vs 15.6%, (RR= 1.65, 95% CI 1.21–2.26), with wound-related morbidity also lower with Rv-TAPP (RR = 1.81, 95% CI 1.25–2.63). Recurrence rates were comparable (1.9% vs 0.5%; RR = 0.95, 95% CI 0.29–3.11). Conclusions: Both robotic extraperitoneal approaches are safe and effective. Rv-TAPP offers shorter operative time and lower wound morbidity without increasing recurrence rates, favouring its use in small to medium-sized or primary ventral defects. Retromuscular repair remains preferred for large or complex hernias, underscoring their complementary roles in contemporary hernia repair.

**Supplementary Information:**

The online version contains supplementary material available at 10.1007/s11701-025-03076-9.

## Introduction

The Rives–Stoppa retromuscular repair has traditionally been regarded as the benchmark technique for open midline ventral hernia reconstruction, offering reliable long-term outcomes and effective restoration of abdominal wall anatomy [1]. Its effectiveness largely derives from placing the mesh within the retromuscular space, which provides wide prosthetic overlap, prevents direct contact with intra-abdominal organs, and has consistently been associated with reduced recurrence rates, fewer wound infections, and improved functional stability of the abdominal wall [2–4].

As minimally invasive surgery evolved, efforts increasingly focused on limiting wound morbidity and shortening hospital stay. Laparoscopic intraperitoneal onlay mesh (IPOM) rapidly became widespread but carries the inherent disadvantage of intraperitoneal mesh positioning, which may lead to long-term issues such as adhesions or visceral erosion [5, 6]. The introduction of robotic platforms has further transformed the field, enabling refined dissection and more advanced reconstructive maneuvers while maintaining the advantages of minimally invasive access. Enhanced three-dimensional visualization, improved dexterity, and stable instrument control allow surgeons to replicate extraperitoneal mesh placement similar to open surgery.

Several robotic retromuscular techniques—such as the transabdominal retromuscular repair (TARUP/TARM) [7] and enhanced-view totally extraperitoneal (eTEP) approaches [8] —apply the Rives–Stoppa principles within the retromuscular compartment. However, these procedures often require crossing the midline with incision and subsequent reconstruction of the posterior rectus sheath, raising concerns about potential alterations to abdominal wall mechanics.

To avoid such disruption while still maintaining visceral separation, the preperitoneal plane offers an alternative extraperitoneal route. This layer can be accessed through a transabdominal ventral TAPP approach [9–11] or via a fully extraperitoneal dissection (PeTEP) [12]. These strategies have gained increasing attention in recent years.

Despite the growing clinical adoption of both planes, direct comparative data between robotic preperitoneal and retromuscular repairs remain limited.This systematic review and meta-analysis therefore aims to quantitatively compare these two robotic approaches with respect to operative performance, intra and postoperative morbidity, surgical-site events, recurrence, and hospital stay, in order to better delineate their respective roles in contemporary ventral hernia surgery.

## Methods

### Data sources and search strategy

A comprehensive search of the peer-reviewed literature was conducted across Medline (PubMed), Embase, Scopus, and the Cochrane Library for studies published between January 1, 2000 and October 20, 2025.

The search strategy included combinations of the following terms: *robotic*, *ventral hernia*, *incisional hernia*, *umbilical hernia*, *epigastric hernia*, *preperitoneal*, *r-TAPP*, *retromuscular*, *retrorectus*, *Rives-Stoppa*, *TAR*, *TARUP*, *posterior component separation*, and *sublay*.

The full PubMed MeSH search string and its adaptations for the other databases are reported in Supplementary Figure S1.

The review followed PRISMA recommendations and AMSTAR II methodological standards [13, 14]. The protocol was prospectively registered in PROSPERO (CRD420251173188). Reference lists of all relevant articles were additionally screened to capture any missing studies.

### Study selection

Two investigators (FB, SL) independently screened titles and abstracts and subsequently reviewed the full texts of potentially eligible articles using Rayyan. Eligibility disagreements were resolved by consultation with a third reviewer (GD).

Studies were eligible if they met the following criteria:

1. randomized or observational comparative design;
2. adult patients with primary or incisional ventral hernia;
3. robotic repair performed either in the preperitoneal or retromuscular plane;
4. reporting of surgical technique and short- and/or long-term outcomes.

No geographical restrictions were applied.

Reviews, editorials, case series with fewer than five patients, and studies focused on alternative minimally invasive approaches were excluded.

Patients undergoing concomitant procedures were not considered, and duplicate data from the same research group were removed.

### Data extraction and methodological appraisal

Two authors independently extracted key study characteristics, including publication year, country, population source, sample size, demographic information, operative variables, postoperative outcomes, length of hospital stay, recurrence data, and follow-up duration.

Study quality was assessed using the Methodological Index for Non-Randomised Studies (MINORS) tool [15].

Risk-of-bias evaluations for each study are summarized in Supplementary Table S1.

Certainty of evidence was synthesized using the GRADE framework [16], classifying each outcome as high, moderate, low, or very low.

Up- or downgrading decisions were based on considerations such as risk of bias, inconsistency, indirectness, imprecision, publication bias, magnitude of effect, and residual confounding.

The GRADE assessment was performed by FB and SL (Supplementary Table S2).

### Assessment of publication bias and sensitivity

Because fewer than ten studies were included, formal tests for publication bias (e.g., funnel plots, Egger’s test) were not undertaken due to their poor reliability in small evidence bases.

Instead, potential small-study effects were explored descriptively by comparing individual study estimates with pooled results.

Robustness of the meta-analytic findings was examined using a leave-one-out sensitivity analysis, sequentially removing each study to identify influential outliers.

### Outcome measures

Primary outcomes.

- operative time.
- overall complications, including differentiation into minor vs. major events.

Complications were graded according to the Clavien–Dindo classification, with Grades I–II considered minor and Grades III–IV considered major.

Secondary outcomes.

- surgical-site events (seroma, hematoma, surgical-site infection).
- length of stay (LOS).
- recurrence.
- 30-day readmission.
- postoperative pain.

### Data synthesis and statistical analysis

Risk ratios (RRs) were used for dichotomous variables.

Continuous variables (e.g., operative time) were synthesized as weighted mean differences using a DerSimonian–Laird random-effects model.

For studies reporting grouped operative time data (e.g., Vargas et al., 2023), means and standard deviations were estimated by assigning the midpoint of each interval and applying standard formulas for grouped data.

Where medians and interquartile ranges were provided, these were converted to mean ± SD using established methods [17].

Between-study heterogeneity was quantified with Cochran’s Q and I² statistics, categorizing I² values as low (< 25%), moderate (25–50%), or substantial (> 50%).

A continuity correction of 0.5 was applied in instances of zero-event cells.

All analyses and forest plots were generated using Python (v3.12) with the *scipy* and *matplotlib* libraries.

A two-sided p value < 0.05 was considered statistically significant.

## Results

### Baseline characteristics

**Fig. 1:**
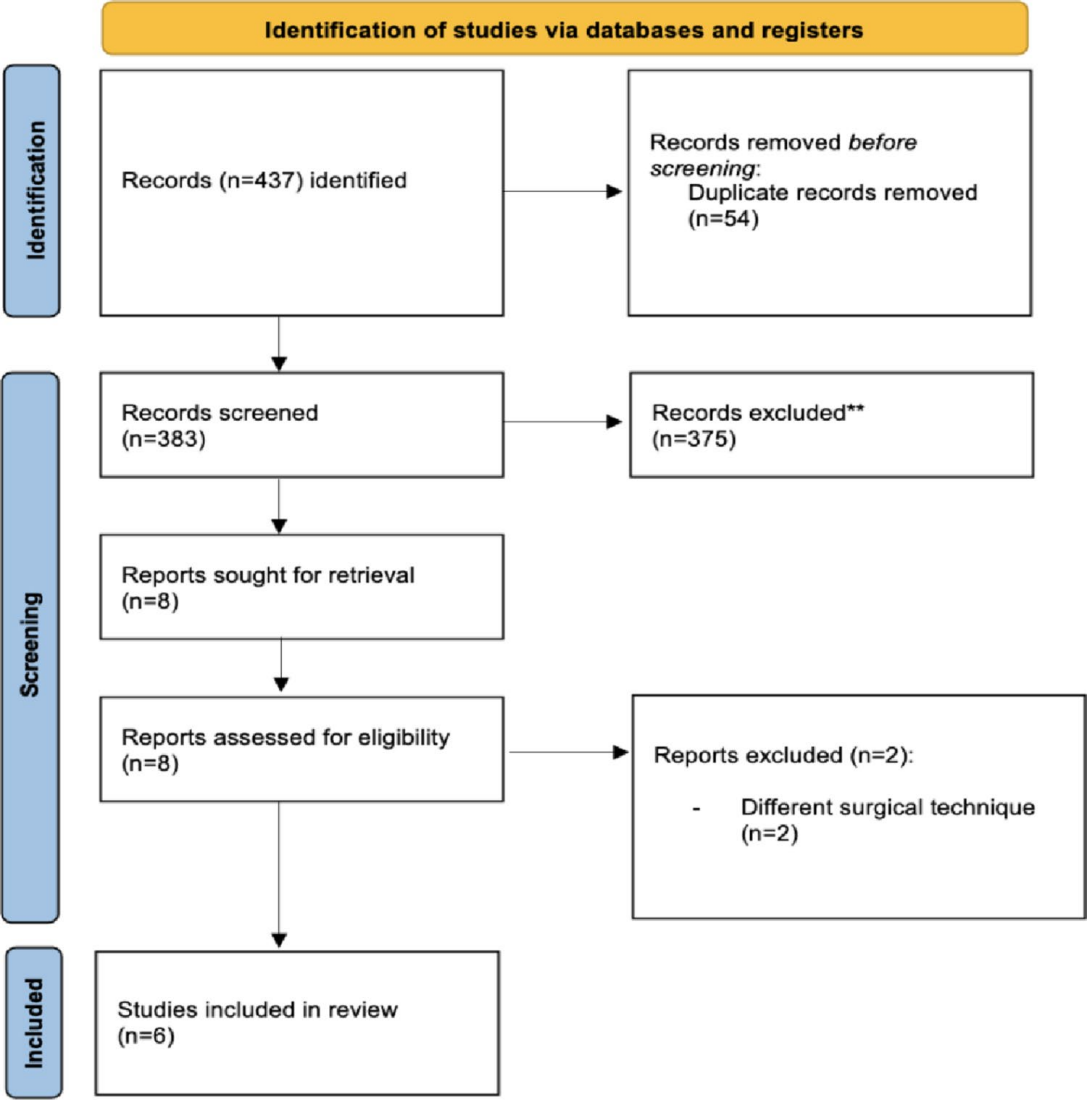
PRISMA flow diagram of selected studies

The flow of study identification and selection is illustrated in Fig. 1. A summary of the main features of the included studies, along with baseline group characteristics, is presented in Table 1.

**Table 1:** Selected studies reporting the preperitoneal vs. retromuscular approach

Study (year)	Country/Setting	Design, study period	Population/Inclusion criteria	Sample size (Rv-TAPP/*r*-Retro)
**Baur et al.**,** 2021**	Switzerland	Retrospective study	Elective midline ventral/incisional hernias	118 (88 Rv-TAPP vs. 30 rRS)
**Kudsi et al.**,** 2022**	USA	Retrospective comparative, 2013–2020	Elective midline ventral/incisional hernias	309 (156 Rv-TAPP vs. 153 rRS + 138 rTAR)
**Vargas et al.**,** 2023**	USA (ACHQC registry, > 150 centers)	Multicenter registry analysis, 2012–2021	Robotic ventral/incisional hernia repairs)	2777 (1829 Rv-TAPP with TAR in 10 patients (0.5%) vs. 948 r-RS with TAR in 131 patients (13.8%))
**Pini et al.**,** 2024**	Switzerland	Retrospective single-center cohort	Primary or incisional umbilical/epigastric hernias	53 (21 Rv-TAPP vs. 32 eTEP)
**Chelliah et al.**,** 2025**	USA	Retrospective multi-arm cohort, 2022–2023	Robotic ventral/incisional hernia repair	243 (199 Rv-TAPP vs. 19 rRS, either TARM or eTEP, and 25 rRS with TAR)
**Alfarawan et al.**,** 2025**	Germany	Retrospective cohort, PSM 1:1, 2023–2024	Adults undergoing robotic ventral or incisional hernia repair	66 (33 Rv-TAPP, with 4 (12.1%) TAR vs. 33 eTEP, with 6 (18.2%) TAR)

**Table 2:** Characteristics of the populations included in the analysis

Study (year)	Age (years), (Rv-TAPP/*r*-Retro)	Female (%), (Rv-TAPP/*r*-Retro)	BMI (kg/m²), (Rv-TAPP/*r*-Retro)	Primary/Incisional (*n*,%), (Rv-TAPP; *r*-Retro)	Defect size (cm/classification), (Rv-TAPP/*r*-Retro)	Defect size (cm^2^), median (IQR), (Rv-TAPP/*r*-Retro)
**Baur et al.**,** 2021**	52.3 ± 13.7/62.1 ± 13.3	25 (28.4)/15 (50.0)	30.7 ± 6.4/29.2 ± 5.4	78 (88.6)/10 (11.3); 11 (36.6)/19 (63.4)	2.3 ± 1.1/4.9 ± 1.1	8.8 ± 9.4/20.1 ± 17.7
**Kudsi et al.**,** 2022**	52.1 ± 14.3/54.6 ± 14.2	53 (34)/52 (34)	31.9 ± 6.2/31.1 ± 6.5	117 (75)/37 (23.7); 108 (70.6)/45 (29.4)	2 (2–3)/4 (3–4)	3.1 (3.1–7.4)/15.7 (11.8–1.8)
**Vargas et al.**,** 2023**	54.0 (43.0–65.0)/57.0 (46.0–66.0)	42.7/52.3	31.5 (27.6–36.0)/31.7 (27.8–36.2)	873 (47.7)/680 (37.2); 187 (19.7)/690 (72.8)	3.0 (2.0–4.0)/6.0 (4.0–11.0)	NR
**Pini et al.**,** 2024**	60.9 (16.6)/57.8 (11.9)	14.3/15.6	29.5 (6.3)/27.1 (5.5)	19 (90.5)/2 (9.5); 25 (78.1)/7 (21.9)	2.5 (0.8)/2.5 (0.9)	NR
**Chelliah et al.**,** 2025**	56 (24–87)/61 (30–84)	24.2/52.3	31 ± 5/30 ± 5	160 (80.4%)/39 (19.6%); 5 (11.4%)/39 (88.6%)	3.8 ± 1.9/12.4 ± 6.3	NR
**Alfarawan et al.**,** 2025**	54 ± 15/58 ± 12	39.4/30.3	30 (8.5)/27 (7.5)	10 (30.3)/23 (69.7); 15 (45.4)/18 (54.5)	W1 19 (59.4)/14 (43.6) W2 8 (25)/7 (21.9) W3 5 (15.6)/11 (34.4)	12 (29)/21 (71)

Across the six retrospective included studies [13–18], a total of 3704 patients underwent robotic ventral hernia repair, of whom 2326 were treated with a preperitoneal approach and 1378 with a retromuscular repair. Baseline demographic and clinical characteristics were broadly comparable between groups, except for hernia and mesh dimensions. The proportion of primary hernias was higher among preperitoneal repairs (*n* = 1257/2326; 54.04%) than retromuscular repairs (*n* = 361/1378; 26.2%). Indeed, the average hernia defect size was 3.0 ± 0.9 cm for preperitoneal approach and 6.3 ± 3.4 cm for retromuscular repairs. Mesh area was reported in all six studies, with consistent differences between approaches. The mean mesh size was 153.6 cm² for preperitoneal repairs and 340.7 cm² for retromuscular repairs, indicating that retromuscular approaches required, on average, a twofold larger mesh surface.

### Meta-analysis

#### Operative time

All six studies reported operative time [13–18]. The pooled median operative time across all included studies was 91 min (IQR 79–119) for preperitoneal repairs and 136 min (IQR 111–170) for retromuscular repairs. Except for one small series [14], all studies consistently showed longer operative times for retromuscular repair.

**Fig. 2:**
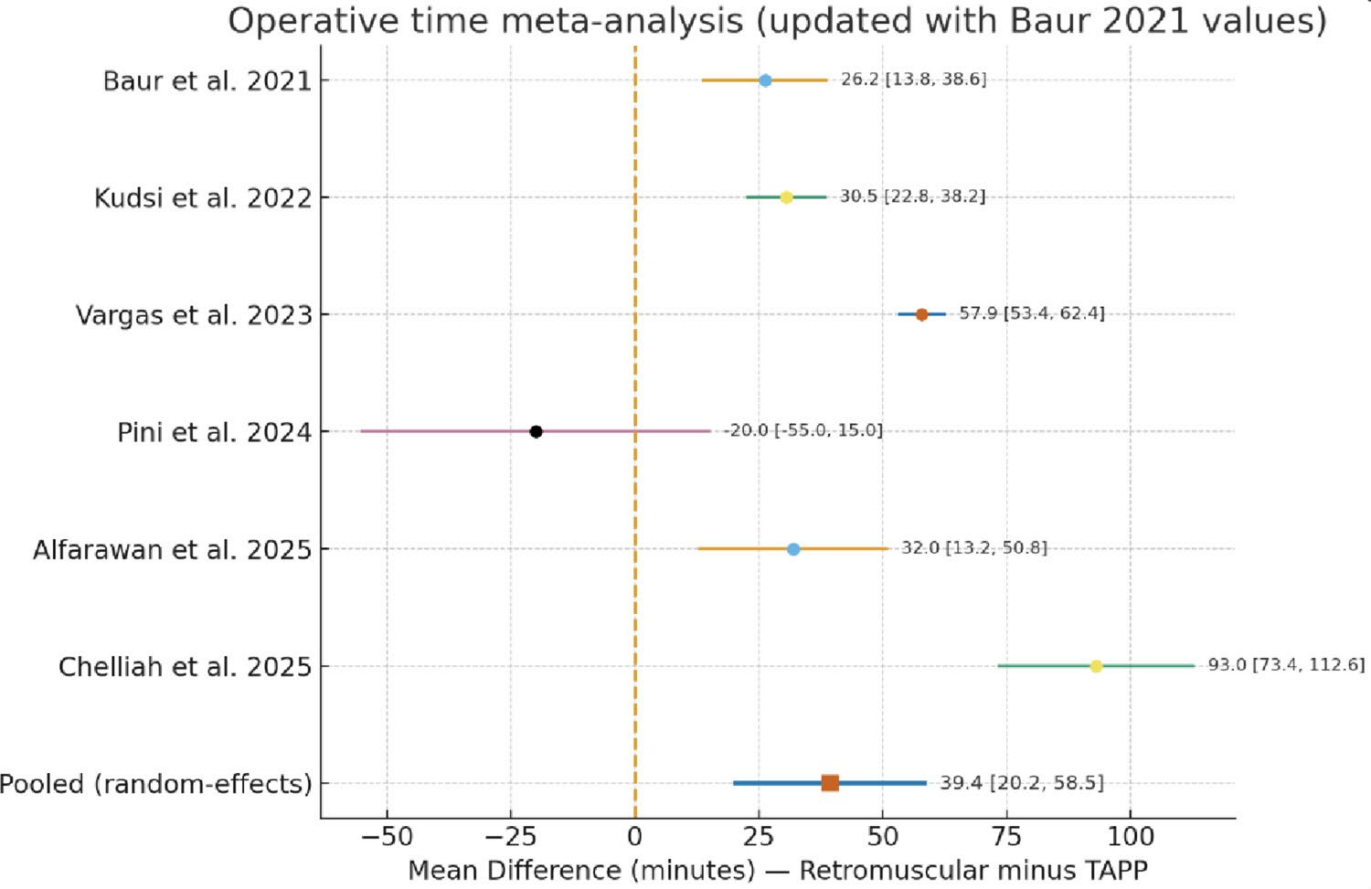
Forest plot of operative time

The pooled random-effects analysis demonstrated a significant overall difference favoring the ventral TAPP approach, with a weighted mean difference (WMD) of + 39.4 min (95% CI + 20.2 to + 58.5, *p* < 0.0001), indicating that retromuscular repairs required approximately forty minutes longer on average (Fig. 2).

Between-study heterogeneity was very high (Q = 79.9, df = 5, *p* < 0.0001; I² = 93.7%; τ² = 470.5).

Nevertheless, the direction of effect was consistent in five of six studies, confirming that operative time is systematically longer in retromuscular repairs.

A leave-one-out sensitivity analysis was performed to assess the influence of each individual study on the pooled estimate for operative time (Supplemetary Figure S2). The weighted mean difference remained statistically significant in all iterations (range + 29.5 to + 47.2 min, all *p* < 0.01), with consistently high heterogeneity (I² > 91%). No single study exerted a disproportionate influence on the direction or significance of the overall effect, supporting the stability of the finding that retromuscular repairs require longer operative time than robotic TAPP.

#### Length of stay

Length of hospital stay (LOS) was reported in most included studies, though the definition of inpatient versus outpatient management varied considerably across series.

Baur et al. reported significantly longer hospitalization after retromuscular repair (2.7 ± 1.7 days) compared with Rv-TAPP (1.5 ± 0.6 days, *p* < 0.001) [17].

In Pini et al., approximately one-third of patients in both groups were treated as outpatients (same-day discharge 28.6% for Rv-TAPP, 31.2% for r-eTEP) [14]. When outpatients were included, the estimated overall LOS was 1.64 ± 1.52 days for Rv-TAPP and 1.79 ± 2.68 days for r-eTEP, showing minimal difference between approaches.

In Vargas et al. [18], most patients were discharged within 24 h, with median LOS = 0 [0–1] days for Rv-TAPP and 1 [0–2] days for retromuscular repair (*p* < 0.05).

Alfarawan et al. [15] found identical median LOS of 2 days (IQR 0) for both eTEP and TAPP, indicating no difference between techniques in that cohort. Given the lack of dispersion data, these two studies were not included in the quantitative pooling but support the overall trend toward short, often overnight or same-day, hospital stay for both approaches.

**Fig. 3:**
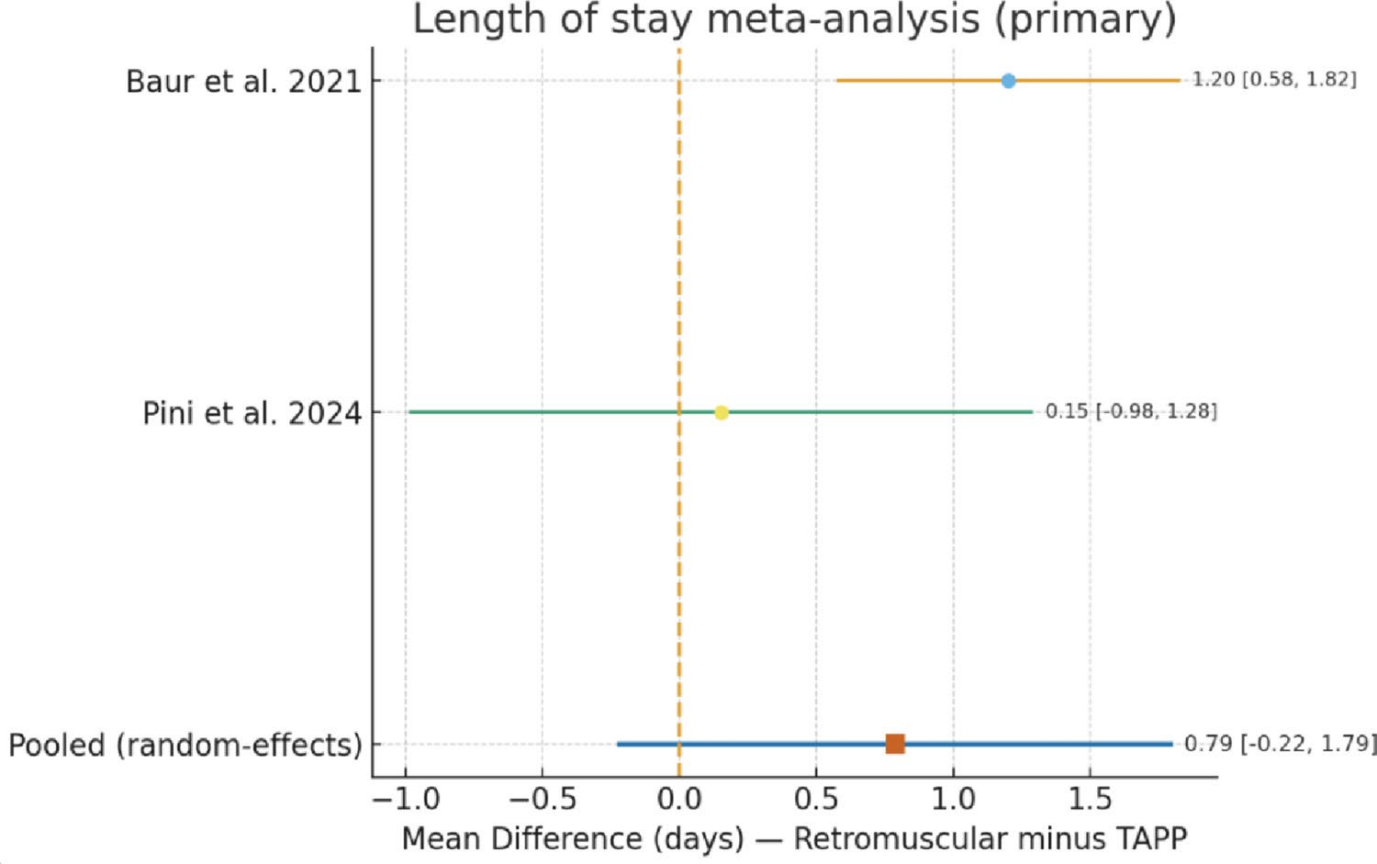
Forest plot of length of stay

A random-effects meta-analysis including Baur 2021 and Pini 2024 showed a nonsignificant tendency toward longer hospitalization after retromuscular repair, with a pooled weighted mean difference (WMD) of + 0.79 days (95% CI − 0.22 to + 1.79, *p* = 0.13) (Fig. 3).

Heterogeneity was moderate (I² = 68%).

#### Postoperative complications

All studies reported overall postoperative complications [13–18]. Overall postoperative complications occurred in 167 of 2326 patients (7.18%) following robotic TAPP or preperitoneal repair, and in 215 of 1378 patients (15.6%) after robotic retromuscular repair. The largest registry analysis [18] showed 6.6% complications after preperitoneal TAPP (121/1829) versus 15.1% after retromuscular repair (143/948; *p* < 0.001). Smaller series were directionally consistent: Chelliah et al. [13] reported 1.0% (2/199) vs. 4.5% (2/44), Pini et al. [14] 2024 0/21 vs. 2/32, and Alfarawan et al. [15] 2025 0/33 vs. 1/33. Kudsi et al. [16], showed 14.1% (22/156) in Rv-TAPP and 19.6% (57/291) in retromuscular (RS with or without TAR). Baur et al. [17] reported 25% (22/88) vs. 33% (10/30).

**Fig. 4:**
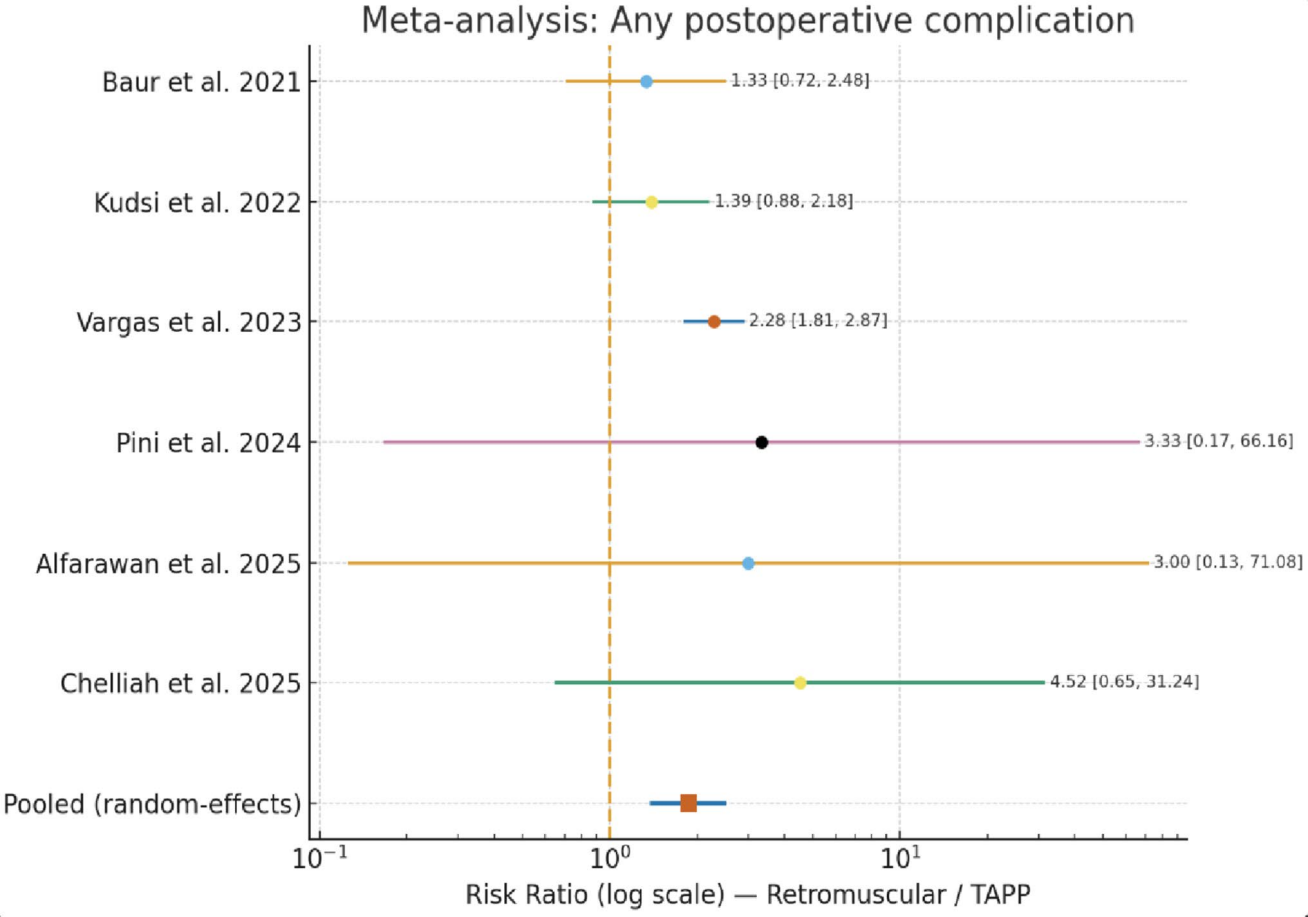
Forest plot of overall postoperative complications

Pooling all six studies with a random-effects model yielded a significantly higher complication risk after retromuscular repair: RR = 1.65 (95% CI 1.21–2.26), z = 3.16, *p* = 0.0016. Heterogeneity was moderate (I² = 43.3%, Q = 8.82, *p* = 0.117; τ² = 0.037). The direction of effect was consistent across studies; zero-event arms (Pini, Alfarawan) were handled with a standard 0.5 continuity correction and did not change the inference (Fig. 4).

**Fig. 5:**
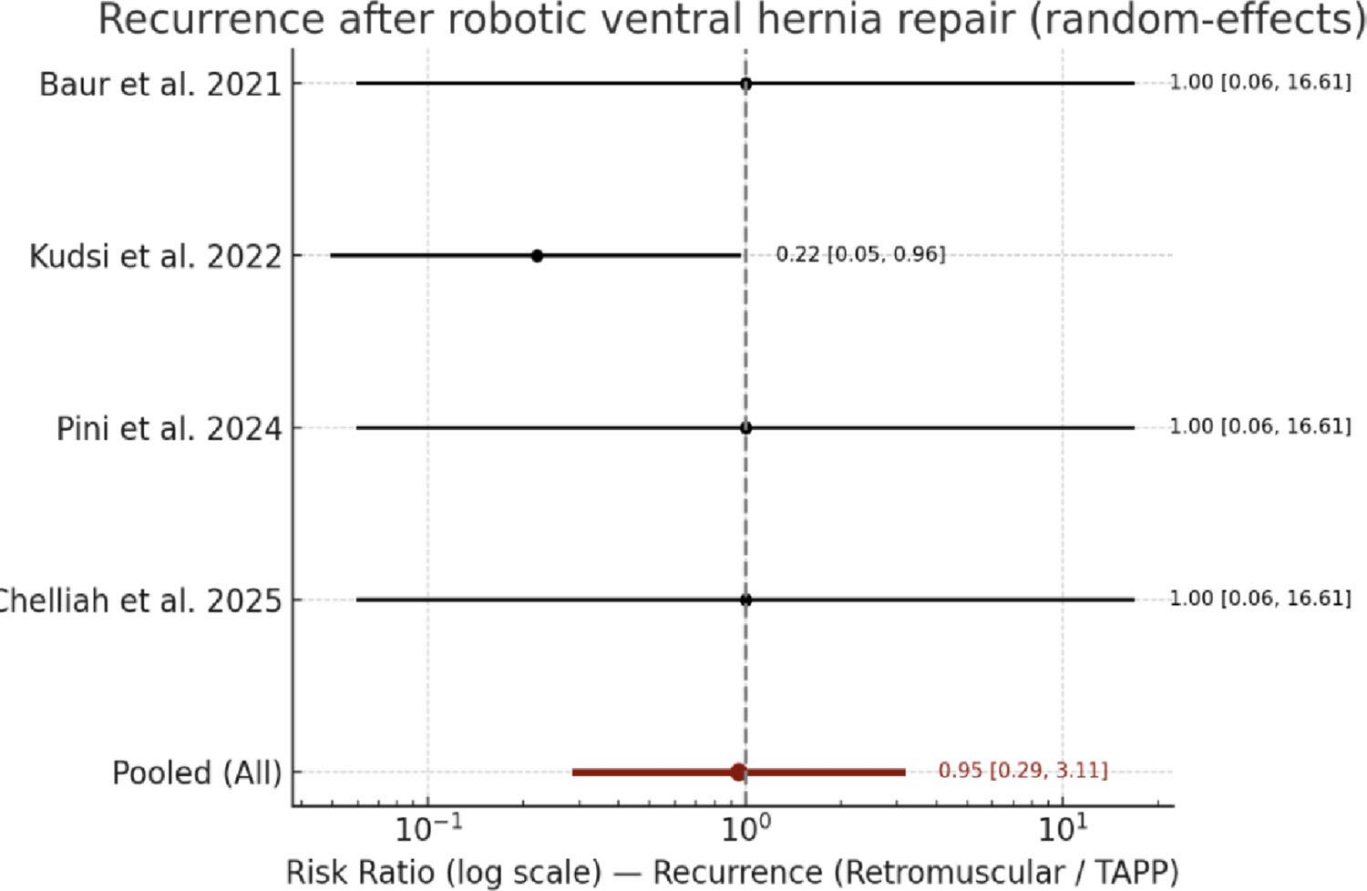
Forest plot of hernia recurrence

Regarding severity grading, formal Clavien–Dindo classification was provided only by Baur et al. [17] and Kudsi et al. [16]. In Baur, the vast majority of events were Grade I (Rv-TAPP *n* = 23; r-RS *n* = 9), with a few Grade II (Rv-TAPP *n* = 2) cases and only two serious events in the retromuscular arm (one Grade III and one Grade IV). No Grade ≥ III complications were reported after TAPP. Similarly, Kudsi’s supplementary analysis confirmed that most complications were mild (Grade I–II; Rv-TAPP *n* = 11; rRS *n* = 14; rTAR *n* = 40) in both groups, while Grade III–IV events were infrequent and largely confined to TAR or more complex retromuscular cases (rRS *n* = 5; rTAR *n* = 7).

#### Recurrence rate

Four studies reported this outcome [13, 14, 16, 17]. Recurrence was a rare event, with a cumulative rate of 1.9% (9/464) after robotic TAPP and 0.5% (2/397) after robotic retromuscular repair.

The pooled random-effects analysis, including four studies reporting this outcome, showed no significant difference between robotic TAPP and robotic retromuscular repair (RR = 0.95, 95% CI 0.29–3.11; I² = 0%).

When restricting the analysis to studies with a mean follow-up of at least 12 months (Kudsi et al. and Chelliah et al.), the pooled estimate remained non-significant (RR = 0.59, 95% CI 0.09–3.93), confirming the absence of differences between techniques over the mid-term follow-up.

#### Wound-related morbidity

Across the three studies reporting these details [14, 16, 17], postoperative seroma and hematoma rates were specifically reported for both Rv-TAPP and retromuscular robotic repairs.

Overall, considering a total of 618 patients (265 undergoing Rv-TAPP and 353 retromuscular repairs), seroma occurred in 23 of 265 patients (8.7%) in the Rv-TAPP group and 26 of 353 patients (7.4%) in the retromuscular group. Hematoma occurred in 5 of 265 patients (1.9%) after Rv-TAPP and 9 of 353 patients (2.5%) after retromuscular repair.

**Fig. 6:**
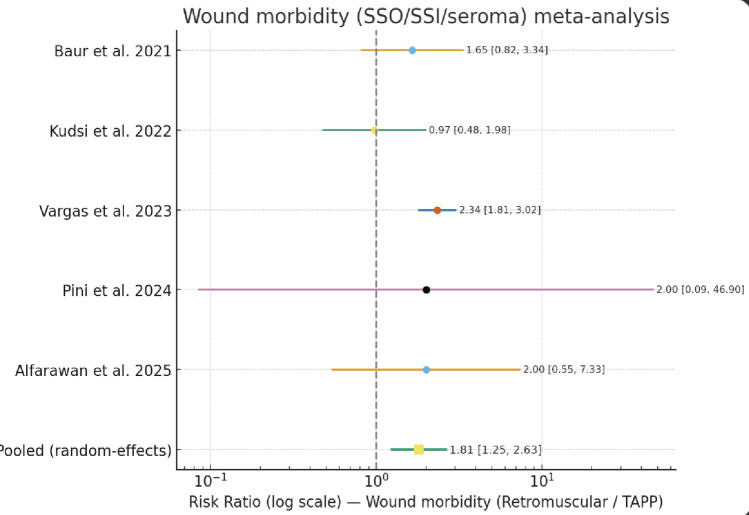
Forest plot of wound morbidity

In terms of overall wound-related complications—including seroma, surgical site infection, or any surgical site occurrence—were reported in 129 of 2127 patients (6.1%) following robotic TAPP or preperitoneal repair, and in 156 of 1334 patients (11.7%) after robotic retromuscular repair. The random-effects meta-analysis across five studies showed a significantly higher risk of wound morbidity with retromuscular approaches (RR 1.81, 95% CI 1.25–2.63; I² 49%) (Fig. 6).

#### Methodological quality of studies

All six included studies were non-randomized comparative analyses, with median MINORS scores of 14 out of 24 (IQR = 1), indicating moderate methodological quality. The main sources of bias were the retrospective design, absence of prospective sample size calculation, and non-equivalent baseline hernia complexity, as retromuscular repairs typically addressed larger or incisional defects. Nevertheless, all studies clearly defined their objectives, used appropriate endpoints, and performed adequate statistical analyses.

According to the GRADE assessment, the overall certainty of evidence ranged from low to very low across outcomes. Evidence was low for operative time, length of stay, and overall complications; low-to-moderate for wound-related morbidity; and very low for recurrence, mainly due to the limited number of events and follow-up duration.

Detailed results of the MINORS quality assessment and the GRADE certainty of evidence are provided in the Supplementary Materials (Tables S1–S2).

## Discussion

This systematic review and meta-analysis synthesizes current evidence comparing robotic preperitoneal ventral TAPP (Rv-TAPP) with robotic retromuscular repair (rRS) for ventral hernia. Across more than 3,700 patients, both techniques were associated with low recurrence rates, short length of stay, and acceptable morbidity, supporting the safety and effectiveness of robotic extraperitoneal repair. However, retromuscular procedures required longer operative time and showed a higher incidence of wound-related complications, while recurrence and overall postoperative outcomes remained comparable between the two approaches.

A major limitation of the available evidence is that r-TAPP was predominantly performed for smaller and primary defects, whereas retromuscular repair—often with TAR—was used for larger or more complex hernias. This substantial baseline imbalance restricts the ability to draw direct comparative conclusions or to generalize r-TAPP outcomes to all defect types.

Within this context, the preperitoneal plane may represent a suitable option for selected patients who would otherwise undergo intraperitoneal or retromuscular repair, as it avoids mesh–visceral contact, preserves midline anatomy, and leaves the retromuscular space available should recurrence occur. However, these potential advantages require confirmation in prospective studies specifically designed to evaluate patient selection across anatomical categories.

Shorter operative time for r-TAPP, consistent with prior reports including Maatouk et al. [19], likely reflects the smaller dissection field and the absence of midline crossover and posterior sheath reconstruction, which are intrinsic to retromuscular repair.

The retromuscular approach requires wide posterior dissection, creation of dead space, and restoration of the posterior sheath, all features that predictably prolong the procedure.

Learning-curve data further contextualize these findings. Cumulative-sum analyses of robotic ventral TAPP have shown time reductions of 10–12 min per procedural phase after approximately 40–50 cases, suggesting that operative efficiency increases once surgeons reliably identify and maintain the correct preperitoneal plane [20].

However, r-TAPP is not inherently simple: preserving an intact peritoneal flap—particularly beyond the fatty trident [21]—is technically demanding in early learning curves [22, 23]. Conversely, TARUP/TARM requires precise anatomical knowledge and confident midline crossover. Thus, time differences likely reflect both anatomical constraints and differing familiarity with each plane across institutions. These considerations are also consistent with the European Hernia Society (EHS) robotic training pathway, which places robotic ventral TAPP early in the learning sequence—together with inguinal r-TAPP and r-IPOM—while reserving retromuscular approaches for later stages [22–24]. This positioning reflects primarily the anatomical progression of difficulty rather than procedural simplicity: even experienced robotic surgeons may find that maintaining an intact peritoneal flap without tears requires advanced tissue handling, whereas retromuscular approaches demand confident midline entry and stable orientation within deeper fascial planes.

Both procedures were associated with short hospital stays (≤ 2 days), reinforcing the benefits of minimally invasive extraperitoneal repair.

However, our analysis identified a 1.6-fold higher risk of wound morbidity after retromuscular repair. This is biologically plausible given the larger retromuscular flap, increased dead space, and disruption of the linea alba required for crossover.

These findings align with Maatouk et al. [19], who reported seroma as the most frequent event after v-TAPP, generally low-grade and conservatively managed. In our analysis, most wound events were minor (Clavien–Dindo I–II), with few serious infections or hematomas.

The reduced disruption of posterior fascial structures in preperitoneal repairs may contribute to lower wound morbidity. Future studies should evaluate whether avoiding crossover translates into measurable improvements in patient-reported outcomes and abdominal wall function (i.e. lower rates of epigastric bulge due to preservation of upper neurovascular bundles and posterior rectus sheath) [25].

Recurrence was uncommon (< 3%) and did not differ significantly between techniques.

Experimental data support the biomechanical validity of both planes: preperitoneal placement provides double-sided tissue contact promoting mesh incorporation, while the retromuscular plane offers broad coverage and robust fixation [26]. A recurring concern raised by critics of the preperitoneal approach is whether a mesh—especially a lightweight polypropylene prosthesis—covered only by a thin peritoneal layer provides adequate protection against adhesion formation or long-term displacement. These theoretical risks deserve acknowledgement; however, the available clinical data do not indicate increased adhesion-related morbidity or mesh migration after Rv-TAPP. Experimental and animal studies also suggest that a well-vascularized peritoneal interface can support stable mesh integration and reduce visceral contact. Nevertheless, high-quality prospective studies with systematic assessment of adhesion-related outcomes are needed to fully clarify the long-term behaviour of preperitoneal prostheses in the ventral domain.

Given the limited follow-up of included studies, equivalence beyond one year remains uncertain. Long-term data are necessary to confirm durability.

From a physiological standpoint, the preperitoneal plane preserves the linea alba and posterior rectus sheath, minimizing disruption of the abdominal core anatomy.

Retromuscular repair, especially when crossover is required, changes the relationship between fascial layers and may theoretically modify abdominal wall mechanics, although this has not been clinically demonstrated [2].

**Fig. 7:**
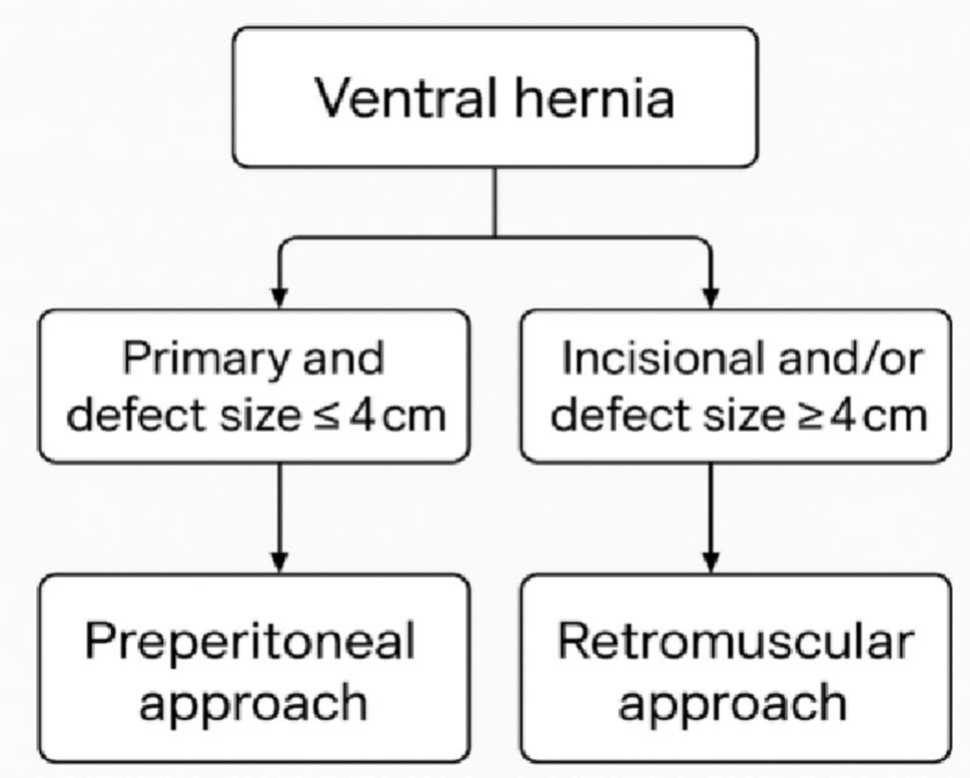
Proposed algorithm for selecting between the preperitoneal and retromuscular approaches

The overall evidence supports a complementary rather than competing role for r-TAPP and rRS.

r-TAPP appears particularly suited for small to medium (smaller than 4 cm) primary defects, as also stated by Kudsi et al. [16], and for selected suprapubic, lateral, and subxiphoid hernias where the peritoneal plane is intact and easily developed (Fig. 7).

Retromuscular repair remains essential for large, complex, or recurrent hernias requiring extensive reconstruction or when the peritoneum is too thin or scarred to support a durable flap.

Preperitoneal extraperitoneal approaches (PeTEP) are gaining traction as they may simplify dissection—starting at the fatty trident and extending laterally—and allow wide mesh overlap [12, 27]. Nonetheless, surgeons performing Rv-TAPP should be proficient in TARUP/TARM as a bailout option.

This meta-analysis is limited by the retrospective design of most included studies and by substantial baseline imbalance, as r-TAPP was generally performed for smaller primary defects, whereas retromuscular repair was reserved for larger and more complex hernias. Extraperitoneal techniques were also not methodologically uniform, with variability in the extent of dissection, mesh size, posterior compartment handling, closure strategies, and drain use—all of which may have influenced operative time and SSO rates (Supplementary Table S3). Follow-up was generally short, functional outcomes were rarely reported, and registry-based datasets lacked sufficient granular detail to stratify results by defect complexity or peritoneal integrity. These factors limit the interpretability of pooled estimates and highlight the need for more standardized prospective studies.

Despite these limitations, the consistency of effect direction across cohorts strengthens confidence in the overall findings.

Prospective randomized studies comparing r-TAPP and retromuscular repair with standardized outcomes and ≥ 24 months of follow-up are needed.

Integrating functional imaging, validated quality-of-life metrics, and cost-effectiveness analysis will be essential to define the true comparative benefit of each plane.

Algorithmic frameworks incorporating defect size, location, and tissue quality may support individualized plane selection in robotic ventral hernia surgery.

## Conclusions

Both robotic preperitoneal (r-TAPP) and retromuscular repairs are effective minimally invasive approaches for ventral hernia repair. Current evidence shows that r-TAPP is associated with shorter operative time and lower wound morbidity, with similar early recurrence compared with retromuscular repair.

Retromuscular techniques remain essential for larger or more complex defects, where broader reconstruction is required. Given the predominantly retrospective nature of the available studies and the baseline differences between groups, these findings should be interpreted cautiously.

Overall, r-TAPP and retromuscular repair should be regarded as complementary options within robotic hernia surgery, with plane selection tailored to defect characteristics, tissue quality, and surgeon expertise.

## Supplementary Information

Below is the link to the electronic supplementary material.

Supplementary Material 1

## Data Availability

No datasets were generated or analysed during the current study.

## Declarations

### PROSPERO ID

CRD420251173188.
