# Correction: Optimizing robotic approach to ventral hernia repair: an updated systematic review and meta-analysis between preperitoneal versus retromuscular repair

**DOI:** 10.1007/s11701-026-03493-4

**Published:** 2026-05-20

**Authors:** Francesco Brucchi, Sara Lauricella, Sofia Esposito, Giampaolo Formisano, Roberto Cirocchi, Richard Sassun, Gianlorenzo Dionigi

**Affiliations:** 1 University of Milan, Via Festa del Perdono, 7, Milan, 20122 Italy; 2 Colorectal Surgery Unit, Fondazione IRCCS Istituto Nazionale Dei Tumori, Milan, 20133 Italy; 3 Department of General, Emergency Surgery and New Technologies, Baggiovara General Hospital, AOU Modena, Modena, Italy; 4 Department of Surgery, Dipartimento di Scienze della Salute, Asst Santi Paolo e Carlo, University of Milan, Milan, Italy; 5 Department of Digestive and Emergency Surgery, “S. Maria” Hospital, Terni, 05100 Italy; 6 Department of Medicine and Surgery, University of Perugia, Perugia, 06129 Italy; 7 Division of Surgery, Istituto Auxologico Italiano, Istituto di Ricovero e Cura a Carattere Scientifico (IRCCS), Milan, Italy; 8 Department of Pathophysiology and Transplantation, University of Milan, Milan, Italy

**Correction: Journal of Robotic Surgery (2025) 20:101**

10.1007/s11701-025-03076-9

In the original version of the article, the author’s name Giampaolo Formisano was incorrectly written as Gianpaolo Formisano.

