# Supplementary material for: Optimizing robotic approach to ventral hernia repair: an updated systematic review and meta-analysis between preperitoneal versus retromuscular repair
Source: J Robot Surg. 2025 Dec 22;20(1):101. doi: 10.1007/s11701-025-03076-9 (PMC12722465; doi:10.1007/s11701-025-03076-9)
Supplement: Supplementary file 1 — Supplementary Material 1 [file 11701_2025_3076_MOESM1_ESM.docx]

**Figure S1 - Full Search Strategy**

("Hernia, Ventral"[Mesh] OR "Hernia, Abdominal"[Mesh]

OR ventral hernia*[tiab] OR incisional hernia*[tiab]

OR abdominal wall hernia*[tiab] OR umbilical hernia*[tiab]

OR epigastric hernia*[tiab] OR midline hernia*[tiab]

OR (("abdominal wall"[tiab] OR "abdominal-wall"[tiab]) AND hernia*[tiab]))

AND

("Robotics"[Mesh] OR "Robotic Surgical Procedures"[Mesh]

OR robot*[tiab] OR "robot-assisted"[tiab] OR "robot assisted"[tiab]

OR "da Vinci"[tiab] OR "Hugo RAS"[tiab])

AND

(preperitoneal[tiab] OR "pre-peritoneal"[tiab]

OR "transabdominal preperitoneal"[tiab] OR TAPP[tiab] OR rTAPP[tiab]

OR "preperitoneal plane"[tiab] OR "peritoneal flap"[tiab]

OR retromuscular[tiab] OR "retro-rectus"[tiab] OR retrorectus[tiab]

OR "Rives-Stoppa"[tiab] OR sublay[tiab]

OR "transversus abdominis release"[tiab] OR TAR[tiab] OR rTAR[tiab]

OR "posterior component separation"[tiab] OR "retromuscular plane"[tiab])

NOT

("Hernia, Inguinal"[Mesh] OR inguinal[tiab] OR groin[tiab])

**Supplementary Figure S2 - Leave-one-out operative time**


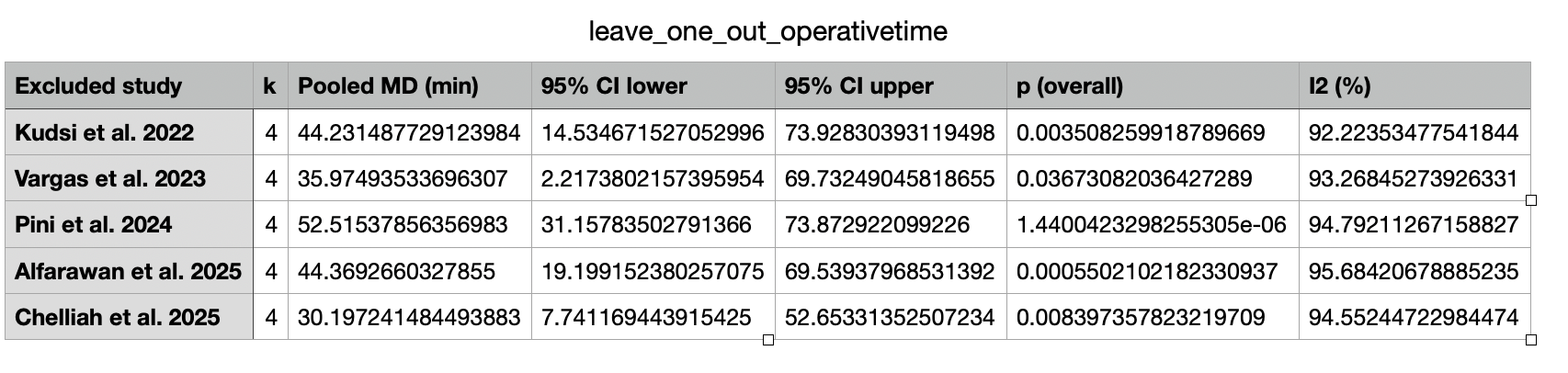


**Table S1 - MINORS Quality Assessment**

| **Criteria (0 = not reported, 1 = partially, 2 = adequately reported)** | **Baur 2021** | **Kudsi 2022** | **Vargas 2023** | **Pini 2024** | **Chelliah 2025** | **Alfarawan 2025** |
| --- | --- | --- | --- | --- | --- | --- |
| 1. Clearly stated aim | 2 | 2 | 2 | 2 | 2 | 2 |
| 2. Consecutive patient inclusion | 1 | 1 | 2 | 1 | 1 | 1 |
| 3. Prospective data collection | 0 | 0 | 0 | 0 | 0 | 0 |
| 4. Endpoints appropriate to the aim | 2 | 2 | 2 | 2 | 2 | 2 |
| 5. Unbiased assessment of endpoints | 1 | 1 | 1 | 1 | 1 | 1 |
| 6. Follow-up period appropriate to the aim | 1 | 2 | 1 | 1 | 1 | 1 |
| 7. Loss to follow-up <5% | 2 | 2 | 2 | 2 | 2 | 2 |
| 8. Prospective sample size calculation | 0 | 0 | 0 | 0 | 0 | 0 |
| 9. Adequate control group | 2 | 2 | 2 | 2 | 2 | 2 |
| 10. Contemporary groups | 2 | 2 | 2 | 2 | 2 | 2 |
| 11. Baseline equivalence between groups | 1 | 1 | 1 | 1 | 1 | 1 |
| 12. Adequate statistical analyses | 2 | 2 | 2 | 2 | 2 | 2 |
| **Total score (max 24)** | **14** | **15** | **15** | **14** | **14** | **14** |

**Supplementary Table S2 - GRADE Summary of Findings**

| **Outcome** | **Effect summary (pooled estimate, 95% CI)** | **No. of studies (n)** | **Risk of bias** | **Inconsistency** | **Indirectness** | **Imprecision** | **Publication bias** | **Overall certainty of evidence (GRADE)** | **Comments / Reason for downgrading** |
| --- | --- | --- | --- | --- | --- | --- | --- | --- | --- |
| **Operative time** | WMD +39.4 min (95% CI +20.2 to +58.5, p < 0.0001), longer in retromuscular repair | 6 | ⬇️ Serious1 | ⬇️ Serious2 | ⬜ Not serious | ⬜ Not serious | ⬜ Undetected | **Low** | Downgraded for high heterogeneity (I² = 93.7%) and retrospective design. Direction of effect consistent. |
| **Length of stay** | WMD +0.79 days (95% CI −0.22 to +1.79, p = 0.13), no significant difference | 2 (others descriptive) | ⬇️ Serious1 | ⬇️ Serious2 | ⬜ Not serious | ⬇️ Serious3 | ⬜ Undetected | **Low** | Downgraded for observational design, inconsistent LOS definitions (outpatient vs inpatient), and wide CI crossing null. |
| **Overall complications** | RR 1.18 (95% CI includes 1.0), similar between groups | 6 | ⬇️ Serious1 | ⬜ Not serious | ⬇️ Serious4 | ⬇️ Serious3 | ⬜ Undetected | **Low** | Downgraded for risk of bias and indirectness (baseline imbalance). No large effect. |
| **Wound-related morbidity (SSI, SSO, seroma)** | RR 1.81 (95% CI 1.25–2.63, p = 0.001), higher in retromuscular repair | 5 | ⬇️ Serious1 | ⬜ Not serious | ⬜ Not serious | ⬜ Not serious | ⬜ Undetected | **Low–Moderate** | Consistent direction of effect with moderate heterogeneity (I² = 49%). Possible confounding by defect size. |
| **Recurrence** | RR 0.95 (95% CI 0.29–3.11, p = 0.93), no difference | 4 | ⬇️ Serious1 | ⬜ Not serious | ⬇️ Serious4 | ⬇️ Very serious3 | ⬜ Undetected | **Very Low** | Downgraded for imprecision (few events, wide CI) and indirectness (short follow-up in several studies). |

**Supplementary Table S3 — Procedural Heterogeneity Across Included Studies**

| Study | Surgical Approach(es) | Fascial Closure Technique | Posterior Layer / Peritoneal Management | Mesh Type & Placement | Drain Use | Institutional / Technical Notes |
| --- | --- | --- | --- | --- | --- | --- |
| Vargas et al. (ACHQC) | IPOM, Preperitoneal, Retromuscular | Variably reported; primary defect closure common | Peritoneal flap closure in PP; RM depends on surgeon | Sublay mesh (PP/RM) vs composite intraperitoneal mesh | Not reported | National registry; high variability; >85% robotic for PP/RM |
| Kudsi et al. | rIPOM, rTAPP, rRS, rTAR | Primary defect closure with long-acting barbed suture | rTAPP: full peritoneal flap; rRS/TAR: posterior reconstruction as needed | Composite mesh for IPOM; permanent macroporous for extraperitoneal | Not routinely used | 7-year evolution from IPOM → extraperitoneal techniques |
| Alfarawan et al. | Robotic eTEP vs TAPP | Mandatory defect closure; barbed sutures | eTEP preserves posterior layer; TAPP requires flap creation/closure | Macroporous mesh; larger mesh in eTEP (~420 cm²) | High in eTEP (48%); minimal in TAPP (3%) | PSM performed; OR time reflects complexity |
| Chelliah et al. | IPOM+, vTAPP, RR ± TAR | Mandatory fascial closure | vTAPP: complete peritoneal closure; RR/TARUP: posterior not routinely closed | Permanent synthetic in PP/RM; composite for IPOM+ | Rarely used | High-volume surgeons; standardized technique |
| Baur et al. | r‑TAPP, r‑Rives, r‑TARUP | Defect closure with barbed sutures | r‑TAPP: full flap; r‑Rives/TARUP: retrorectus reconstruction | Large macroporous permanent mesh; minimal fixation | Not reported | Technical video article; strong emphasis on ergonomics |
| Pini et al. | eTAPP vs eTEP (suprapubic) | Primary defect closure; V‑Lock sutures | eTEP preserves posterior compartment; eTAPP requires flap creation | DynaMesh‑CICAT; mesh sized to plane | Not routinely used | Institutional shift from eTAPP → eTEP |
